# Supplementary material for: Chemicals orchestrate reprogramming with hierarchical activation of master transcription factors primed by endogenous Sox17 activation
Source: Commun Biol. 2020 Oct 30;3:629. doi: 10.1038/s42003-020-01346-w (PMC7603307; doi:10.1038/s42003-020-01346-w)
Supplement: Supplementary file 1 — Supplementary Information [file 42003_2020_1346_MOESM1_ESM.pdf]

Supplementary Fig. 1

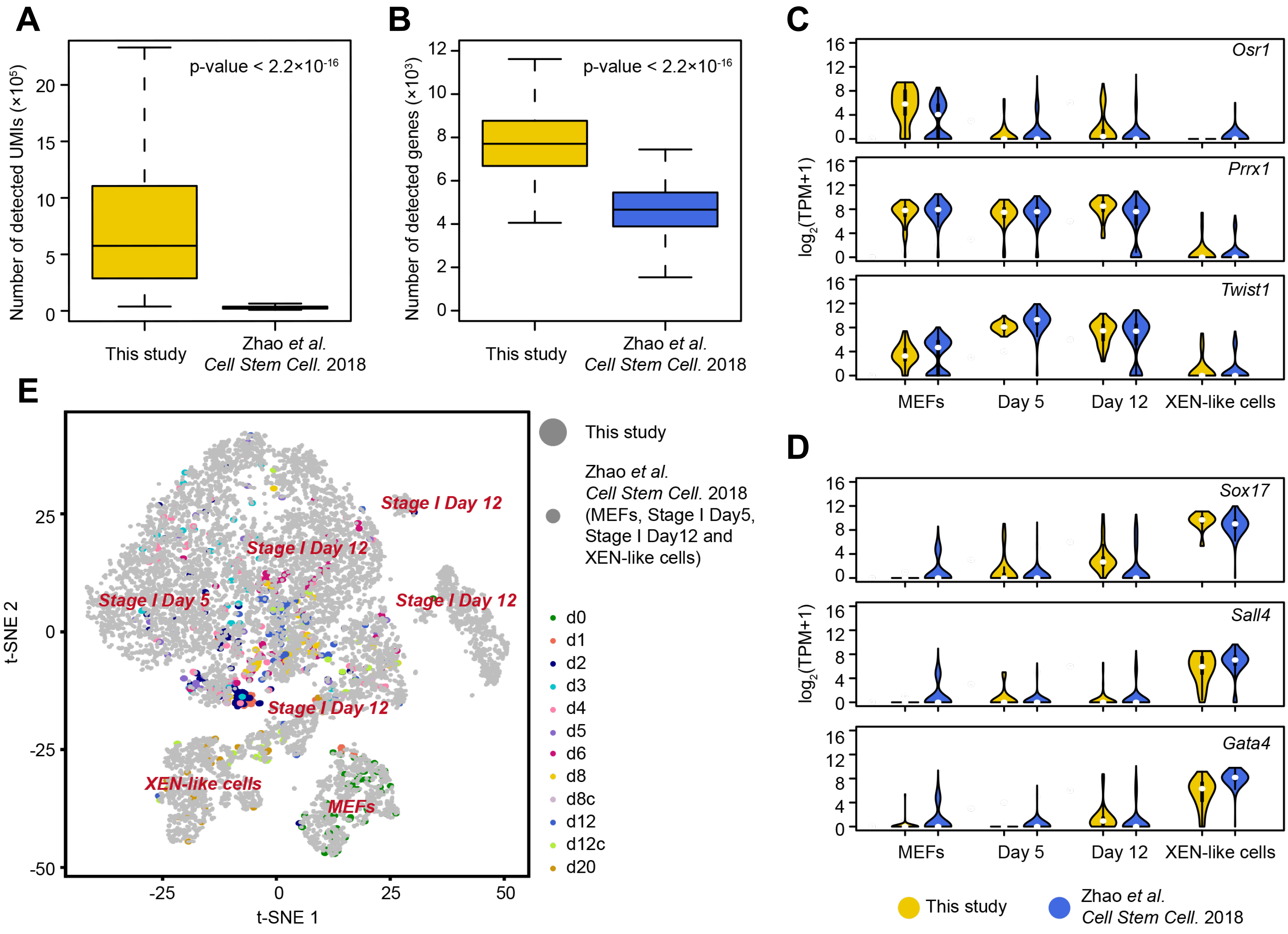

Supplementary Fig. 1

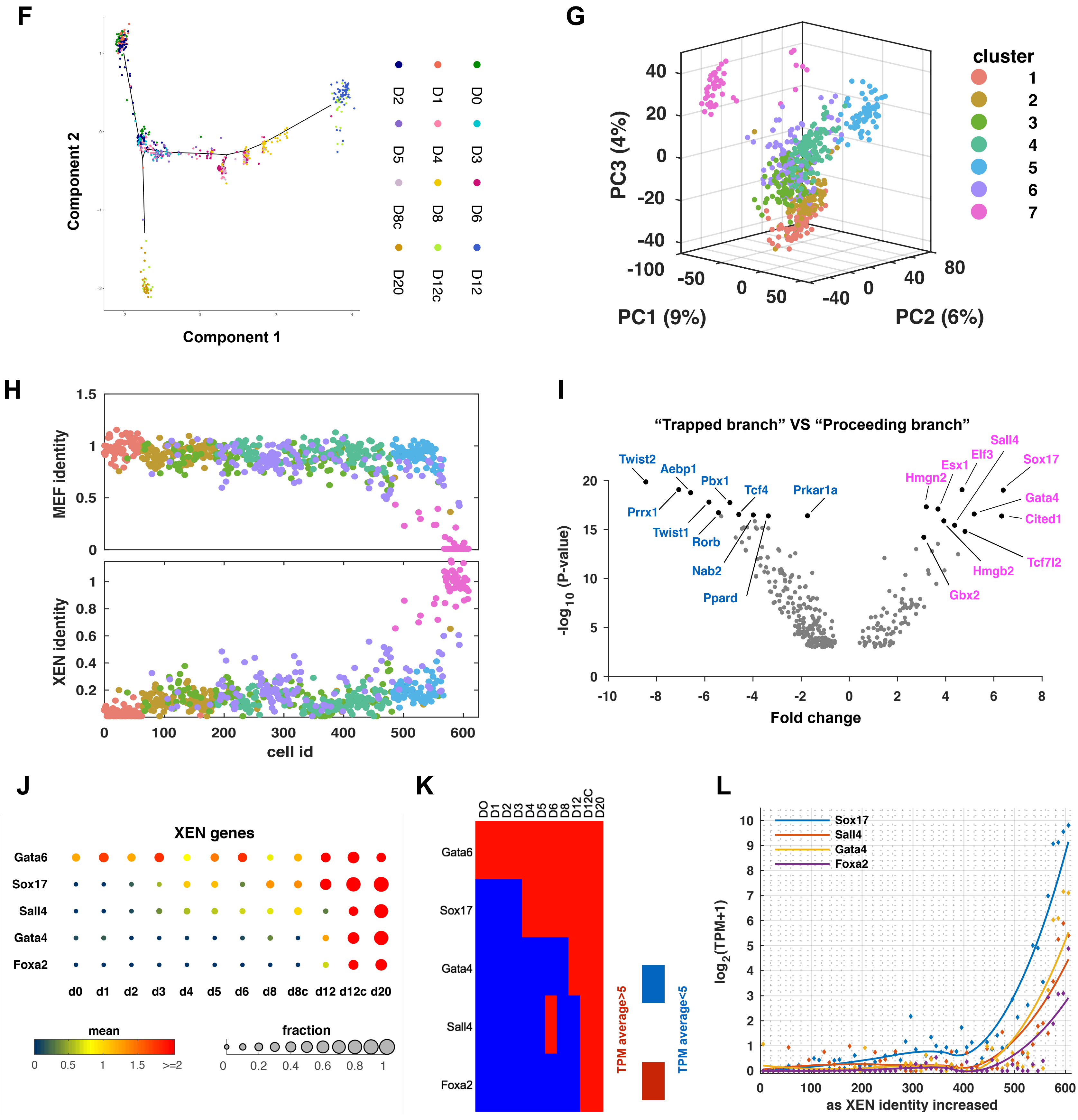

Supplementary Fig. 1

A-B. Number of detected UMIs (A) and genes (B) comparing with data in Zhao *et al. Cell Stem Cell.* 2018.

C-D. Expression level of MEF (C) and XEN (D) master genes comparing with data in Zhao *et al. Cell Stem Cell.*

E. t-SNE of gene expression integrated from data in this study and in Zhao *et al. Cell Stem Cell.* 2018.

F. Trajectory reconstruction of all single cells throughout chemical reprogramming by pseudo-time analysis.

G. PCA map for single cells with clustering information.

H. MEF and XEN identity of each cluster.

For each cell on the XEN reprogramming path, the similarity to bulk RNA-seq from either MEFs or XEN-like cells as calculated using quadratic programming.

I. Differentially expressed genes in the trapped and proceeding branch.

J. Expression of XEN genes at the indicated time points. Color key: Log_2(TPM+1).

K. Average expression of XEN master genes at different time points. Red, average TPM>5; Blue, average TPM<5.

L. Up-regulated XEN master genes when the XEN identity increased (analyzed by single-cell RNA-seq).

Supplementary Fig. 2

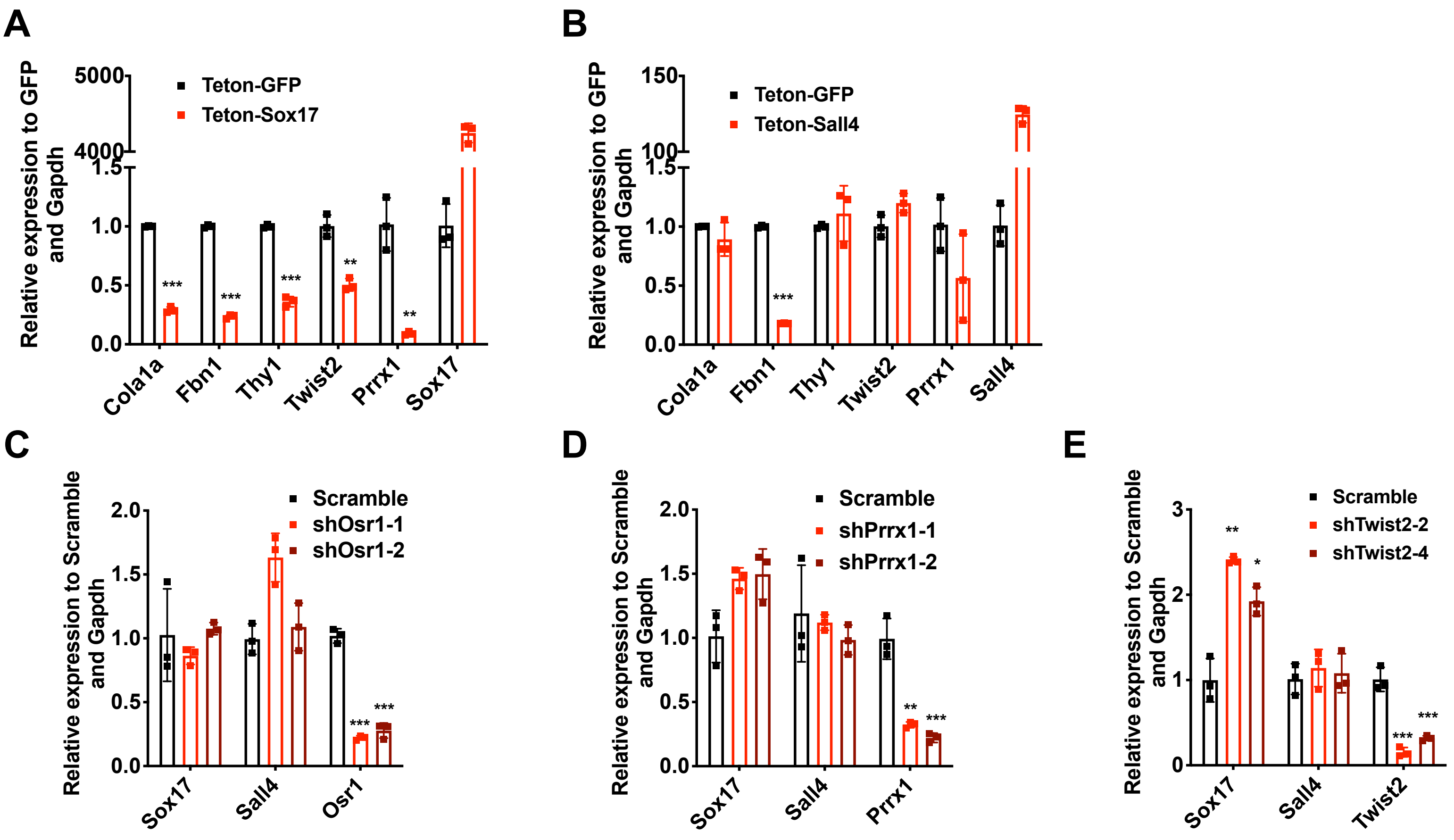

Supplementary Fig. 2

A-B. Relative MEF gene expression induced by C6FAE on day 12 with the overexpression of *Sox17* and *Sall4* (n=3).

C-E. Relative XEN gene expression induced by C6FAE on day 12 with the knockdown of *Osr1*, *Prrx1*, and *Twist2* (n=3).

Significance was assessed compared with the controls using a one-tailed Student's t test. ***$p < 0.001$; **$p < 0.01$; *$p < 0.05$.

Supplementary Fig. 3

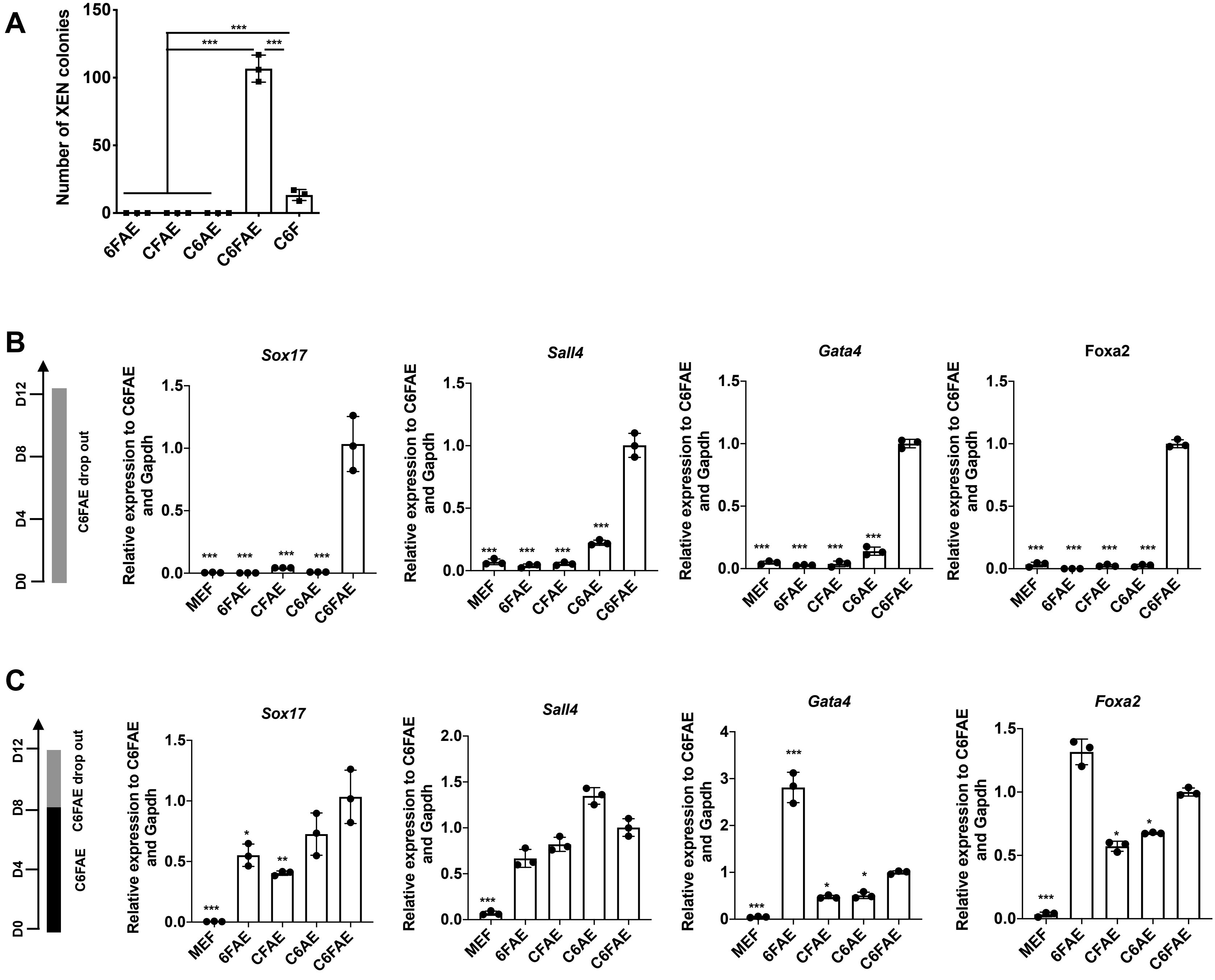

Supplementary Fig. 3

A. Numbers of XEN colonies induced by 6FAE, C6AE, CFAE, C6F and C6FAE at day 12 (n=3).

B. Relative expression of XEN master genes on day 12 after removal of C, 6 or F from day 0 (n=3).

C. Relative mRNA levels of XEN master genes on day 12 after removal of C, 6 or F from day 8 (n=3).

Significance was assessed compared with the controls using a one-tailed Student's t test. ***$p < 0.001$; **$p < 0.01$; *$p < 0.05$.

Supplementary Fig. 4

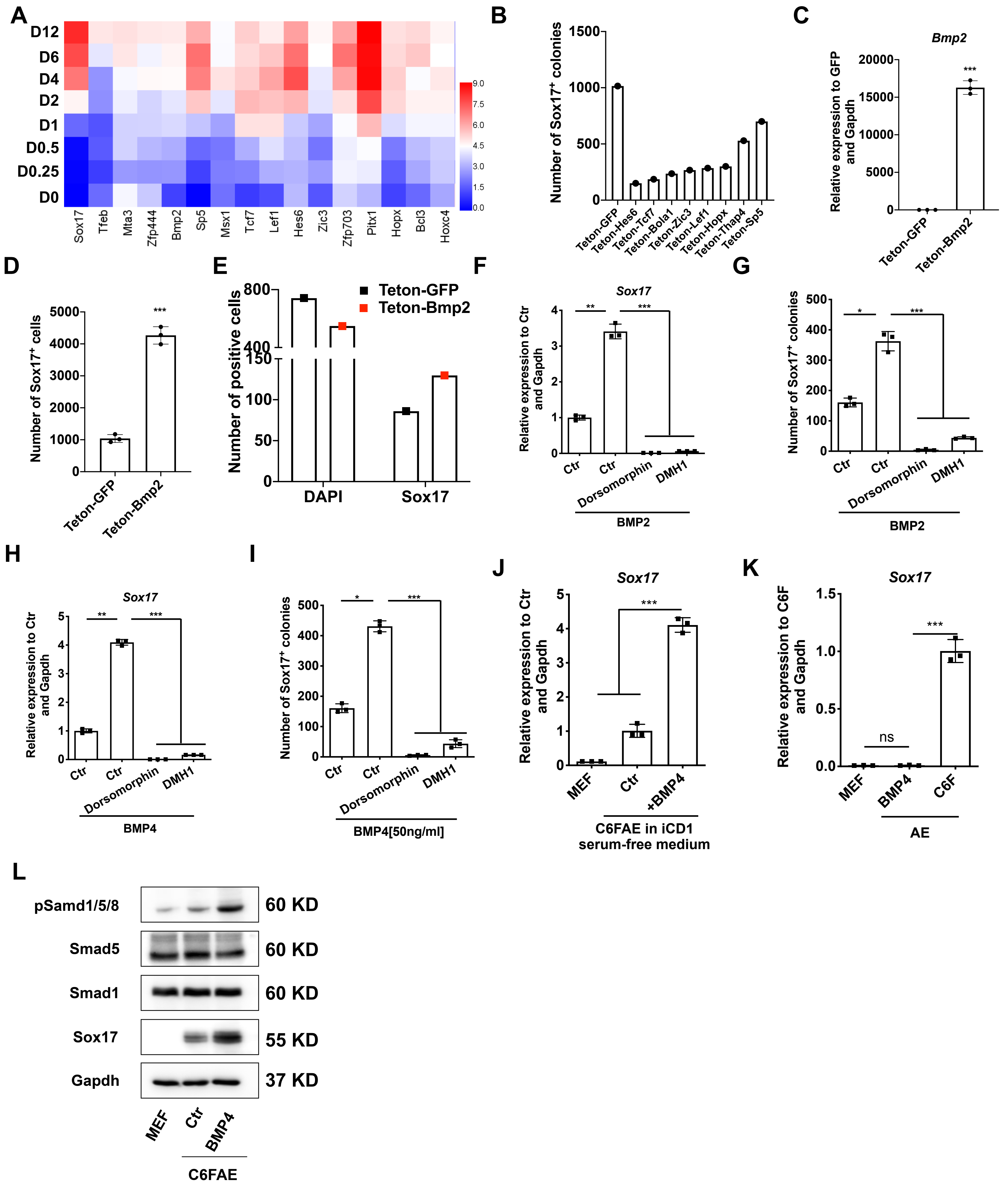

Supplementary Fig. 4

A. Up-regulated genes before the activation of *Sox17* (analyzed by bulk RNA-sequencing).

B. Number of Sox17 positive cells on day 6 with the overexpression of early activated genes.

C. Relative expression of *Bmp2* after overexpression (n=3).

D. Number of Sox17 positive cells on day 6 with the overexpression of *Bmp2* (n=3).

E. Numbers of nucleus and Sox17 positive cells with 6 days overexpression of *Bmp2*.

F. Relative expression of *Sox17* after adding BMP2 with or without DMH1 on day 4 (n=3).

G. Number of Sox17 positive colonies after adding BMP2 with or without DMH1 on day 6 (n=3).

H. Relative expression of *Sox17* after adding BMP4 with or without DMH1 on day 4 (n=3).

I. Number of Sox17 positive colonies after adding BMP4 with or without DMH1 on day 6 (n=3).

J. Relative expression of Sox17 after 6 days treatment with BMP4 on the basis of C6FAE in iCD1 serum-free medium (n=3).

K. Relative expression of Sox17 after 6 days treatment with BMP4 or C6F on the basis of AE (n=3).

L. Effect of BMP4 on inducing Sox17 expression and phosphorylation of Smad1/5/8. Cells treated with cocktails for day 6 were analyzed by western blot.

Significance was assessed compared with the controls using a one-tailed Student's t test. ***$p < 0.001$; **$p < 0.01$; *$p < 0.05$.

Supplementary Fig. 5

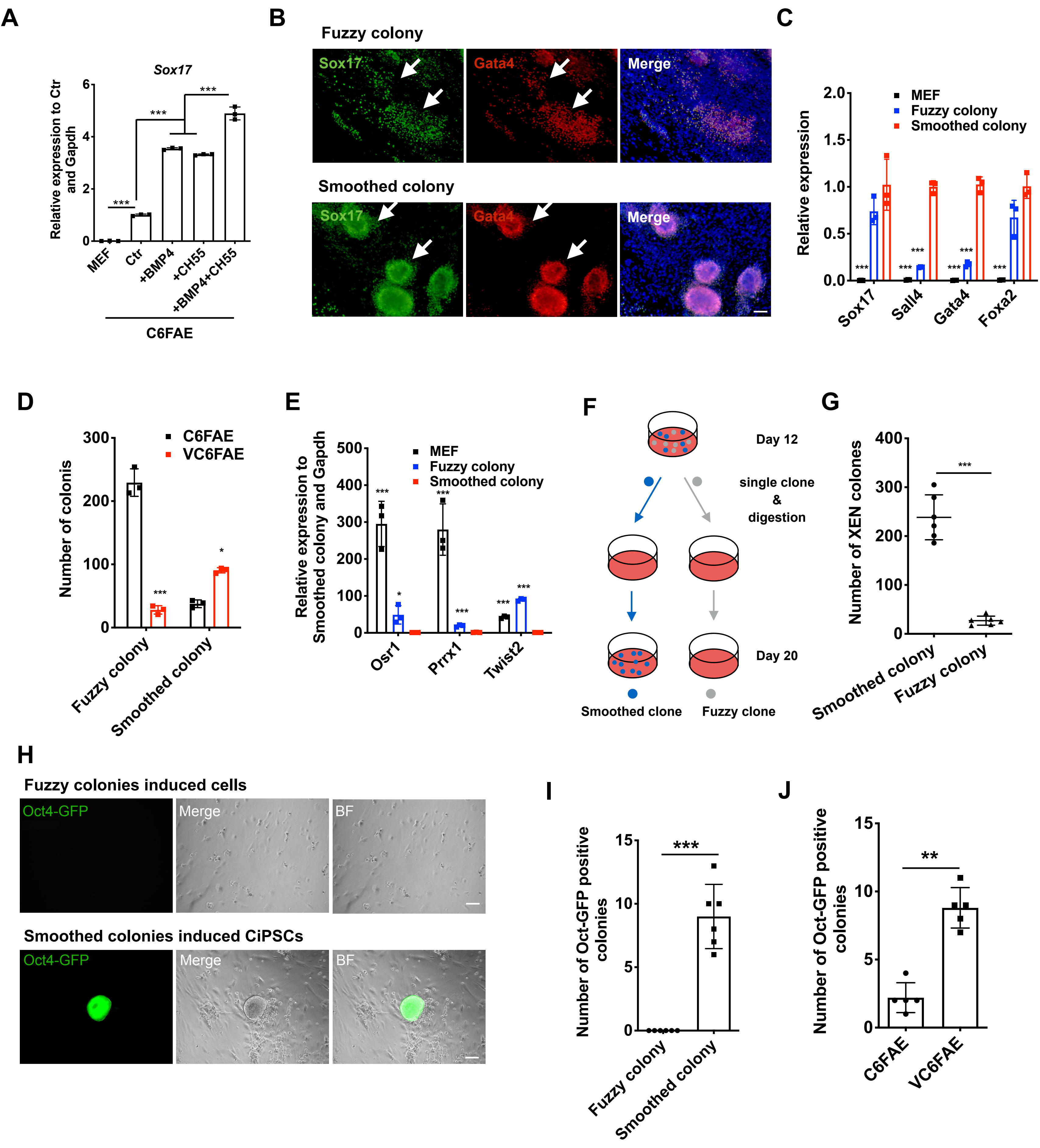

Supplementary Fig. 5

A.Relative expression of Sox17 induced by CH55, BMP4 and CH55+BMP4 on the basis of C6FAE at day 4 (n=3).

B. Morphology and XEN maker gene expression in fuzzy and smoothed colonies. Scale bar, 100 μm.

C. Relative mRNA expression of XEN master genes in fuzzy and smoothed colonies induced by C6FAE on day 12 (analyzed by RT-qPCR, n=3).

D. Numbers of fuzzy colonies and smoothed colonies per well of 12-well plate induced in different chemical combinations on day 12 (n=3).

E. Relative mRNA expression of MEF master genes in fuzzy and smoothed colonies induced by C6FAE on day 12 (analyzed by RT-qPCR, n=3).

F. Schematic of cell passage of fuzzy and smoothed colony.

G. Number of XEN colonies produced from fuzzy and smoothed colony after passage(n=6).

H. Oct4-GFP positive colonies induced from smoothed colonies and cells induced from fuzzy colonies. Scale bar, 100 μm.

I. Numbers of Oct4-GFP positive colonies induced from fuzzy and smoothed colonies (n=6).

J. Numbers of Oct4-GFP positive colonies induced by C6FAE and VC6FAE (n=5).

Significance was assessed compared with the controls using a one-tailed Student's t test. ***$p < 0.001$; **$p < 0.01$; *$p < 0.05$.

Supplementary Figure-blot images

Gapdh

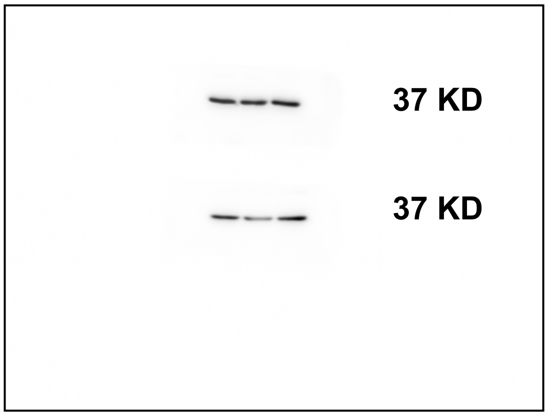

P-Smad1/5/8

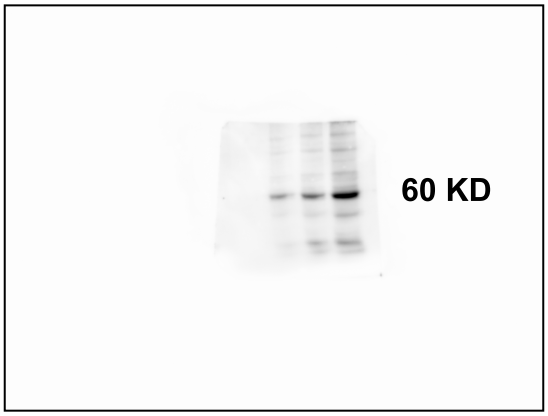

Sox17

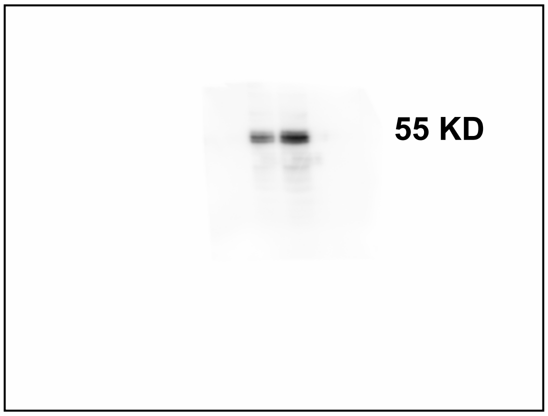

Smad1

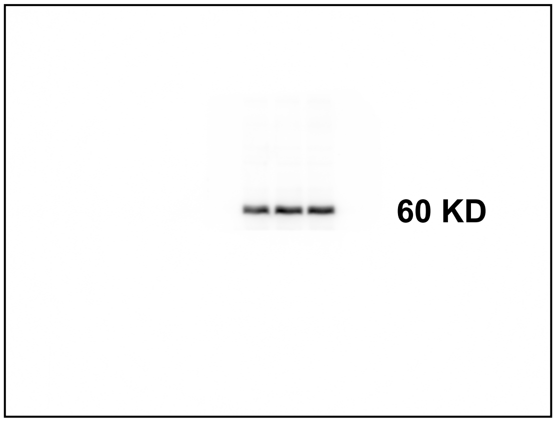

Smad5

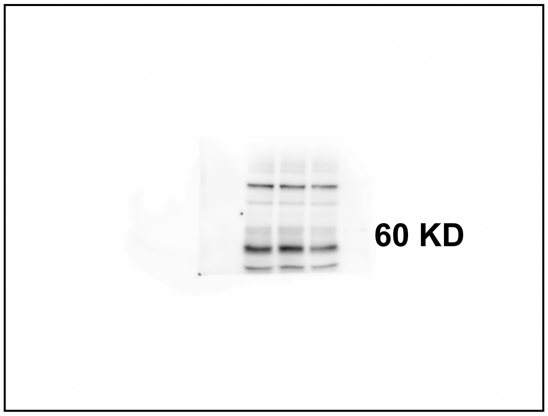

Original pictures of all blot images in Supplementary Fig. 4L.
