# Supplementary material for: Chemicals orchestrate reprogramming with hierarchical activation of master transcription factors primed by endogenous Sox17 activation
Source: Commun Biol. 2020 Oct 30;3:629. doi: 10.1038/s42003-020-01346-w (PMC7603307; doi:10.1038/s42003-020-01346-w)
Supplement: Supplementary file 2 — Description of Additional Supplementary Files [file 42003_2020_1346_MOESM2_ESM.docx]

Description of Additional Supplementary Files

Supplementary Data 1: Source data underlying plots shown in figures.

Supplementary Data 2: Primers of RT-qPCR in this study.
